# Stroke increases motor output variability during maximal voluntary isometric contractions of knee extensor muscles in adults

**DOI:** 10.14814/phy2.70840

**Published:** 2026-03-22

**Authors:** Zhilun Zhou, Stephanie C. Wolfe, Brian D. Schmit, Matthew J. Durand, Sandra K. Hunter, Allison S. Hyngstrom

**Affiliations:** ^1^ Department of Physical Therapy Marquette University Milwaukee Wisconsin USA; ^2^ Department of Kinesiology University of Wisconsin‐Madison Madison Wisconsin USA; ^3^ Department of Interprofessional Studies Carroll University Waukesha Wisconsin USA; ^4^ Joint Department of Biomedical Engineering Marquette University & Medical College of Wisconsin Milwaukee Wisconsin USA; ^5^ Department of Anesthesiology Medical College of Wisconsin Milwaukee Wisconsin USA; ^6^ Department of Physical Medicine and Rehabilitation Medical College of Wisconsin Milwaukee Wisconsin USA; ^7^ Cardiovascular Center Medical College of Wisconsin Milwaukee Wisconsin USA; ^8^ School of Kinesiology University of Michigan Ann Arbor Michigan USA

**Keywords:** inconsistency, knee extensors, motor output variability, stroke, unsteadiness

## Abstract

Greater motor output variability (MOV, inconsistency and unsteadiness) during submaximal tasks post stroke is associated with poorer motor performance. However, little is known about MOV during maximal tasks and relationships with clinical measures of motor performance among stroke survivors. This study determined inconsistency (across discrete attempts) and unsteadiness (within a single attempt) during knee extension maximal voluntary isometric contractions (MVICs) in stroke survivors and neurotypical controls. Forty‐three stroke survivors (28 female) and 31 age‐matched neurotypical controls (15 female) performed a minimum of five knee extension MVICs with paretic and non‐paretic legs (stroke) or dominant leg (controls). Inconsistency was calculated as coefficient of variation between maximal torque values of each MVIC. Unsteadiness was calculated as the average from coefficient of variation of the torque values during each MVIC. The paretic leg of stroke survivors had greater MVIC inconsistency (7.61 ± 4.31% vs. 4.94 ± 2.77%, *p* = 0.014) and unsteadiness (7.00 ± 3.16% vs. 3.44 ± 1.68%, *p* < 0.001) than the dominant leg of neurotypical controls. Greater MVIC inconsistency (*r*_*s*_ = −0.374, *p* = 0.016) and unsteadiness (*r*_*s*_ = −0.445, *p* = 0.004) of stroke survivors' paretic leg were associated with lower Fugl‐Meyer Assessment‐Lower Extremity motor scores. Stroke increases MOV during a single session of MVIC measurements with multiple attempts and is associated with poorer clinical measures of motor performance.

## Graphical abstract

Compared with neurotypical controls, stroke survivors had greater motor output variability (inconsistency and unsteadiness) during a single session of maximal voluntary isometric contractions of the paretic knee extensor muscles, highlighting the importance of using multiple attempts when assessing maximal muscle strength in stroke survivors.

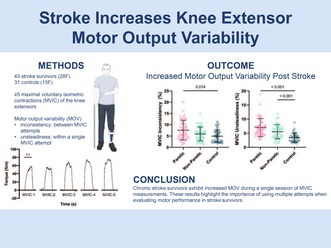

## 1 INTRODUCTION

Clinicians and researchers measure maximal muscle strength as part of stroke survivors' motor function evaluation and use that as a biomarker for their rehabilitation progress. An understudied factor that could potentially affect the accuracy of maximal muscle strength measurements in this population is motor output variability (MOV), which is defined as unintentional variations in performance during voluntary muscle contractions (Christou, 2011). In research, 3 attempts are usually adopted when testing maximal muscle strength in neurotypical individuals (Hunter et al., 2008). Greater MOV post stroke can cause an increase in the number of attempts needed to capture true maximal muscle strength. There are two dimensions of MOV: inconsistency (variability across several discrete attempts) and unsteadiness (variability within a single attempt) (Yacoubi & Christou, 2024). Although inconsistency, to some extent, is desirable in scenarios favoring a large repertoire of responses (e.g., reacting to unanticipated perturbations while ambulating), greater MOV is typically associated with impaired motor performance (Christou, 2011; Christou et al., 2003, 2007; Christou & Enoka, 2011; Harris & Wolpert, 1998; Lodha et al., 2016) especially during anticipated tasks that require precision and fine control of the movements (e.g., eating with utensils). Thus, minimizing MOV and improving motor accuracy is of great importance in stroke evaluation and rehabilitation.

Older neurotypical adults exhibit increased MOV compared with their younger counterparts (Christou, 2011; Christou et al., 2003; Christou & Enoka, 2011; Lodha et al., 2016) and they are typically slow in their execution of tasks to avoid errors and potential consequences such as falling (Hortobágyi & DeVita, 1999). Related to the current study, few studies have demonstrated that MOV might also be increased in people with neurological conditions. Chow and Stokic (Chow & Stokic, 2011) as well as Hyngstrom and colleagues (Hyngstrom et al., 2014) showed that unsteadiness was greater in both the paretic and non‐paretic leg muscles of subacute (Chow & Stokic, 2011) and chronic stroke survivors (Hyngstrom et al., 2014) versus neurotypical controls. Davis et al. showed that people with multiple sclerosis also had greater unsteadiness with their leg muscles compared with neurotypical controls (Davis et al., 2020). Further, the greater unsteadiness from these studies was associated with poorer performance in walking or balance tests (Chow & Stokic, 2011; Davis et al., 2020; Hyngstrom et al., 2014). However, these studies only examined unsteadiness in submaximal contractions, where contraction intensity was ≤50% of maximum. Although sharing the same descending corticospinal tract, sensory feedback from the ascending pathways may be utilized differently during submaximal versus maximal contractions to inform and adjust motor commands. For example, during submaximal contractions, participants rely heavily on visual inputs to instruct fine control of the movements, whereas during maximal contractions (typically without visual feedback), participants may rely more on the proprioceptive and other sensory inputs. Also, the calculation of unsteadiness during submaximal contractions was based on three or fewer attempts. Therefore, the results of these studies may not be generalizable to various daily tasks that require repetition such as climbing the stairs. In addition, activities of daily living for neurotypical individuals usually involve submaximal contractions of the muscles; however, maximal effort may be essential and required for stroke survivors who are weaker in general to complete activities that require high power output such as standing up from a seated position.

To the best of our knowledge, no studies have investigated inconsistency and unsteadiness in stroke survivors during maximal voluntary isometric contraction (MVIC) attempts using a dynamometer, a standardized method to measure maximal muscle strength in laboratory settings. The primary goal of this study was to determine inconsistency (across discrete MVIC attempts) and unsteadiness (within a single MVIC attempt) during knee extension MVICs in stroke survivors and neurotypical controls. We hypothesized that stroke survivors have greater MVIC inconsistency and unsteadiness than neurotypical controls. A secondary goal was to compare potentiated twitch (*Q*_tw_) of the knee extensor muscles between stroke survivors and neurotypical controls. The *Q*_tw_ measures primarily reflect the electrically elicited response from downstream of the neuromuscular junction without inputs from the neural drive, which may provide potential mechanistic insights into the increased MVIC variability in stroke survivors (neural and/or muscular in origin). A third goal was to compare MVIC measurements (torque output, inconsistency, and unsteadiness) between the first and last 3 attempts for stroke survivors and neurotypical controls to characterize within‐session performance across repeated attempts. The fourth goal was to determine relationships between MVIC variability and clinical measures of motor performance in stroke survivors. We hypothesized that greater MVIC variability occurs in stroke survivors with worse motor function.

## 2 MATERIALS AND METHODS

### 2.1 Participants

Participants were recruited using a REDCap stroke database from the Medical College of Wisconsin (PRO00026783), advertisements, and word of mouth. Inclusion and exclusion criteria are listed in Table 1. The present data were baseline measurements collected as part of an ongoing clinical trial (NCT04038697). The study was conducted in accordance with the Declaration of Helsinki. All procedures were approved by Marquette University Institutional Review Board (HR‐1812027206). A written informed consent was obtained from each participant before any study activities.

**TABLE 1:** Inclusion and Exclusion Criteria.

	Inclusion criteria	Exclusion criteria
Stroke survivors	≥18 years of age Able to give informed consent ≥6 months post diagnosis of unilateral cortical or subcortical stroke Residual lower limb paresis	Chronic low back or hip pain Substance abuse in the last 5 years Head trauma in the last 5 years or head trauma with lasting effects Comorbid neurological disorders Uncontrol medical condition Any condition where resisted leg contractions are contraindicated (e.g., musculoskeletal pain/injury) Inability to follow 2‐step commands
Neurotypical controls	≥18 years of age Able to give informed consent	History of stroke Chronic low back or hip pain Substance abuse in the last 5 years Head trauma in the last 5 years or head trauma with lasting effects Comorbid neurological disorders Uncontrol medical condition Any condition where resisted leg contractions are contraindicated (e.g., musculoskeletal pain/injury) Inability to follow 2‐step commands

### 2.2 Clinical assessments

The same licensed physical therapist performed all the clinical assessments. The 10‐meter walk test was used to measure self‐selected walking speed (Bohannon, 1997). Participants were allowed to use assistive devices if needed during the 10‐m walk test. The motor portion of the Fugl‐Meyer Assessment‐Lower Extremity (FMA‐LE, maximum score: 34) was used to assess lower extremity motor impairments in stroke survivors (Fugl‐Meyer et al., 1975).

### 2.3 Torque measurements

#### 2.3.1 Experimental set‐up

Torque generated by knee extensor muscles was measured using a System 4 Pro dynamometer (Biodex Medical Systems, Shirley, NY). Stroke survivors were tested on both their paretic and non‐paretic legs. For consistency, the paretic leg was tested first for each stroke survivor. Neurotypical controls were tested on their dominant leg, which was determined by self‐reported leg to use when kicking a ball. Participants were seated and securely strapped in the Biodex chair with the hip joint flexed at 85° and knee joint flexed at 90°. The center of the knee joint of the testing leg was aligned with the Biodex torque sensor. The leg attachment for isometric contraction was strapped to the testing leg approximately 3 cm above the lateral malleolus. Torque signals from the Biodex dynamometer were collected using custom‐written LabVIEW (NI, Austin, TX) programs through a PCI data acquisition board (NI, Austin, TX) with a 500 Hz low‐pass filter and sampled at 1 kHz.

#### 2.3.2 Maximal voluntary isometric contraction (MVIC)

To measure maximal strength of the knee extensor muscles, participants were asked to perform a minimum of five attempts of MVICs with strong verbal encouragement. In the case that the 5th MVIC attempt produced the highest torque, additional MVIC attempts were performed. The instruction was to kick and hold against the leg attachment as hard as possible for 5 s. A minimum of 1‐min rest was given between each attempt to avoid potential neuromuscular fatigue. The highest MVIC torque value in Newton meters (Nm) among all MVIC attempts from each participant was reported.

#### 2.3.3 Potentiated twitch (*Q*_tw_)

Q_tw_ was measured via electrical stimulation‐induced contractions with a DS7A constant current stimulator (Digitimer, Welwyn Garden City, Hertfordshire, UK) as previously described (Booth et al., 1997) on the paretic leg of stroke survivors and the dominant leg of neurotypical controls. Electrical stimulation was applied via two rectangular (5 × 10 cm) electrodes (Reserv, Hudson, OH). After locating the midpoint between the anterior superior iliac spine and the superior part of the patella, the two electrode pads were placed 2.5 cm proximally and distally to the aforementioned midpoint over the skin of the anterior thigh (Zhou et al., 2025). Twitches were elicited using single square‐wave stimulation pulses (400 V, 200 μs). The stimulation current was gradually elevated until no further increase in twitch torque was observed. Then a supramaximal electrical stimulation at 110% of the current eliciting the highest twitch torque was applied to ensure maximal twitch responses. To measure *Q*_tw_, participants were asked to perform another three to five MVICs, and the supramaximal electrical stimulation was triggered within a 5‐s time window after the completion of each MVIC. Because *Q*_tw_ was assessed via electrical stimulation when participants were at rest, it primarily provides information distal to the neuromuscular junction in the absence of volitional effort, from which we can draw inferences regarding neural or muscular mechanisms.

### 2.4 Experimental protocol

Following informed consent, each participant was invited to the laboratory for two sessions at least 24 h apart. During session 1, participants were familiarized with the Biodex dynamometer and techniques to measure MVIC and *Q*_tw_. Clinical assessments were performed first during session 2, followed by MVIC and *Q*_tw_ measurements.

### 2.5 Data processing

Torque measurements were processed offline. Prior to analysis, baseline corrected torque data was low‐pass filtered at 10 Hz using a zero‐phase 4th order Butterworth filter. The maximal torque value during each MVIC (not followed by electrical stimulation) and *Q*_tw_ measurement was used to calculate inconsistency (coefficient of variation between the maximal torque values) across discrete attempts of MVIC and *Q*_tw_.

$$\text{Inconsistency}=\frac{{\mathrm{SD}}_{\text{across}-\text{attempt maximal torque}}}{{\text{Mean}}_{\text{across}-\text{attempt maximal torque}}}\times 100\%$$

**FIGURE 1:**
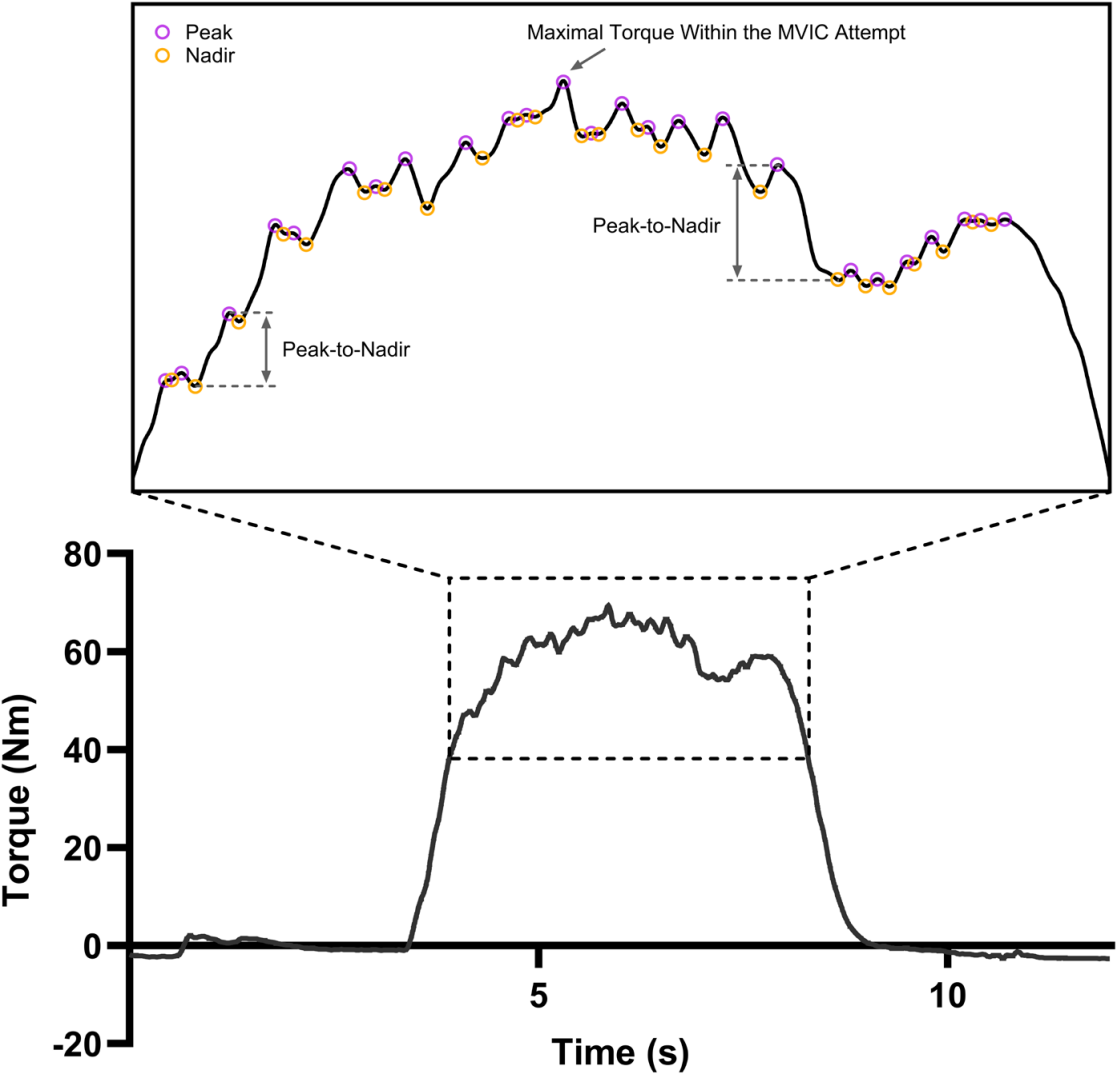
An example of how peak‐to‐nadir fluctuations within each maximal voluntary isometric contraction (MVIC) attempt were calculated.

The actual holding phase of each MVIC (not followed by electrical stimulation) was used to calculate torque unsteadiness within a single MVIC attempt. To determine the actual holding phase of each MVIC, a custom‐written MATLAB (MathWorks, Natick, MA) program was developed to identify all the peak and nadir points within the 5‐s holding phase (Figure 1), which were visually appraised. The first peak point with a torque value higher than 50% of the maximal torque value within that MVIC attempt was assigned as the start of the actual MVIC holding phase, and the last peak point was assigned as the end of the actual MVIC holding phase. The average of the coefficient of variation of torque values during each MVIC attempt for each participant was reported.

$$\text{Unsteadiness}=\frac{{\mathrm{SD}}_{\text{torque during the MVIC holding phase}}}{{\text{Mean}}_{\text{torque during the MVIC holding phase}}}\times 100\%$$

To further investigate torque unsteadiness during each MVIC attempt (not followed by electrical stimulation), we used the aforementioned MATLAB program to calculate the difference in torque value between every adjacent peak and nadir point as shown in Figure 1, we counted the number of peak‐to‐nadir fluctuations that were more than 5% and 10% of the maximal torque value within that MVIC attempt and used that as another indicator of torque unsteadiness during a single MVIC attempt.

To explore the performance over time, we looked at the changes in MVIC measurements (torque output, inconsistency, and unsteadiness) across attempts, specifically between the first 3 and last 3 MVIC attempts (not followed by electrical stimulation).

### 2.6 Statistical analyses

All statistical analyses were performed using IBM SPSS Statistics 28 (IBM, Armonk, NY) and significance levels were set to 0.05. Data are presented as mean ± SD. For each variable, normality and homogeneity of variance were assessed using the Shapiro–Wilk test and the Levene's test, respectively. Nonparametric statistical analyses were performed because all variables violated the assumption for normal distribution and/or equal variance. The Mann–Whitney *U* test was performed to detect differences in participant characteristics between stroke survivors and neurotypical controls. Separate Kruskal‐Wallis tests were performed to detect differences in MVIC, MVIC inconsistency, MVIC unsteadiness, and peak‐to‐nadir fluctuations during a single MVIC attempt among the three testing leg groups (stroke survivors: paretic and non‐paretic legs; neurotypical controls: dominant leg). Post hoc pairwise comparisons with Bonferroni correction were performed if the Kruskal–Wallis test result was significant. The Mann–Whitney *U* test was performed to detect differences in *Q*_tw_ inconsistency between the paretic leg of stroke survivors and the dominant leg of neurotypical controls. Separate Wilcoxon Signed‐Rank tests were performed to detect differences in MVIC measurements (torque output, inconsistency, and unsteadiness) between the first 3 and last 3 MVIC attempts for all three groups. Separate Spearman's rank correlations were made between MVIC MOV measurements and clinical measures of motor performance in stroke survivors.

## 3 RESULTS

### 3.1 Demographics

Forty‐three stroke survivors (28 female) and 31 neurotypical controls (15 female) completed the study. Demographic information is shown in Table 2. There was not a significant difference in age between stroke survivors and neurotypical controls (*U* = 617, *p* = 0.587). Stroke survivors had a higher body mass index (29 ± 6 kg/m^2^ vs. 25 ± 4 kg/m^2^, *U* = 381, *p* = 0.008) and a slower self‐selected walking speed (0.70 ± 0.36 m/s vs. 1.42 ± 0.20 m/s, *U* = 1278, *p* < 0.001) than neurotypical controls. The average FMA‐LE motor score in this cohort of stroke survivors was 23 (maximum score: 34), indicating moderate lower extremity motor impairments.

**TABLE 2:** Demographics.

	Stroke	Control
Age, years	61 ± 12	58 ± 15
Sex	F (28) M (15)	F (15) M (16)
Height, m	1.66 ± 0.10	1.73 ± 0.12
Weight, kg	79 ± 18	76 ± 19
Body mass index^a^, kg/m^2^	29 ± 6	25 ± 4
Paretic (stroke)/Dominant (control) side	L (25) R (18)	L (3) R (28)
Type of stroke	I (20) H (15) U (8)	‐
Time since stroke onset, years	8 ± 8	‐
FMA‐LE motor score, /34	23 ± 6	‐
Self‐selected walking speed^a^, m/s	0.70 ± 0.36	1.42 ± 0.20

*Note*: Data are presented as mean ± SD.

Abbreviations: FMA‐LE, Fugl‐Mayer Assessment‐Lower Extremity; H, Hemorrhagic; I, Ischemic; U, Unknown.

^a^ Significant differences between stroke survivors and neurotypical controls at *p* < 0.05.

### 3.2 MVIC

**FIGURE 2:**
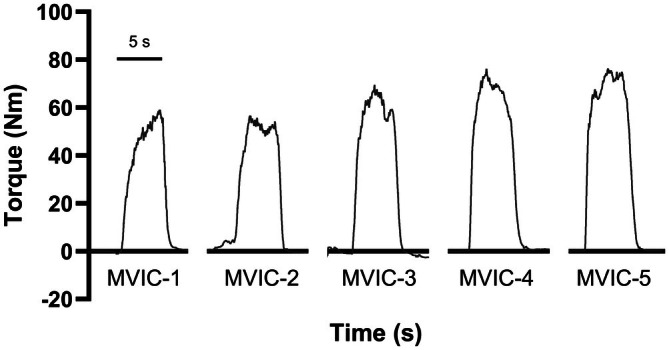
Representative torque traces of five discrete maximal voluntary isometric contraction (MVIC) attempts with knee extensor muscles for a 51‐year‐old male stroke survivor.

**FIGURE 3:**
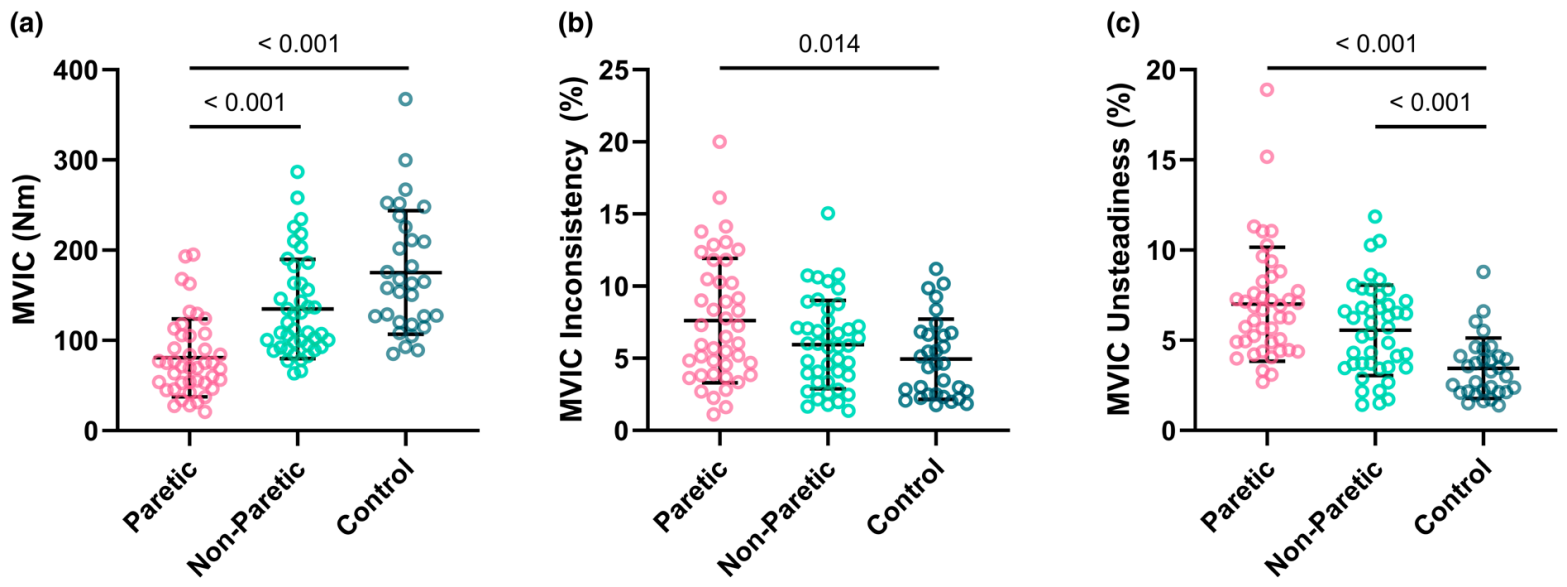
(a) Maximal voluntary isometric contraction (MVIC). The highest MVIC torque value among all MVIC attempts not followed by electrical stimulation from each participant was reported. The paretic leg of stroke survivors had a lower MVIC than their non‐paretic leg and the dominant leg of neurotypical controls. There was no significant difference in MVIC between the non‐paretic leg of stroke survivors and the dominant leg of neurotypical controls. (b) MVIC inconsistency (coefficient of variation between maximal torque values of each MVIC attempt not followed by electrical stimulation). The paretic leg of stroke survivors had greater MVIC inconsistency than the dominant leg of neurotypical controls. No significant differences in MVIC inconsistency were detected between the non‐paretic leg of stroke survivors when compared with their paretic leg or the dominant leg of neurotypical controls. (**c**) MVIC unsteadiness (an average of the coefficient of variation of torque values during each MVIC attempt not followed by electrical stimulation). Both the paretic and non‐paretic legs of stroke survivors had greater MVIC unsteadiness than the dominant leg of neurotypical controls. No significant differences in MVIC inconsistency were detected between the paretic and non‐paretic legs of stroke survivors.

Representative torque traces of discrete MVIC attempts for a stroke survivor are illustrated in Figure 2. Significant differences in MVIC among the three testing leg groups were detected (*H* (2) = 44, *p* < 0.001). Further, as shown in Figure 3a, the paretic leg (80.68 ± 43.09 Nm) of stroke survivors had a lower MVIC than their non‐paretic leg (134.93 ± 54.96 Nm, *p* < 0.001) and the dominant leg of neurotypical controls (175.25 ± 68.44 Nm, *p* < 0.001). There was no significant difference in MVIC between the non‐paretic leg of stroke survivors and the dominant leg of neurotypical controls (*p* = 0.078).

### 3.3 MVIC inconsistency

Significant differences in MVIC inconsistency across discrete attempts among the three testing leg groups were detected (*H* (2) = 8.112, *p* = 0.017). Further, as shown in Figure 3b, the paretic leg of stroke survivors had greater MVIC inconsistency than the dominant leg of neurotypical controls (7.61 ± 4.31% vs. 4.94 ± 2.77%, *p* = 0.014), but no significant differences were detected between the non‐paretic leg (5.95 ± 3.06%) of stroke survivors when compared with their paretic leg (*p* = 0.338) or the dominant leg of neurotypical controls (*p* = 0.505).

### 3.4 MVIC unsteadiness

Significant differences in MVIC unsteadiness within a single attempt among the three testing leg groups were detected (*H* (2) = 32.256, *p* < 0.001). Further, as shown in Figure 3c, both the paretic leg (7.00 ± 3.16%, *p* < 0.001) and non‐paretic leg (5.55 ± 2.51%, *p* < 0.001) of stroke survivors had greater MVIC unsteadiness than the dominant leg of neurotypical controls (3.44 ± 1.68%), but no significant differences were detected between the paretic and non‐paretic legs of stroke survivors (*p* = 0.096).

### 3.5 Peak‐to‐nadir fluctuations

**FIGURE 4:**
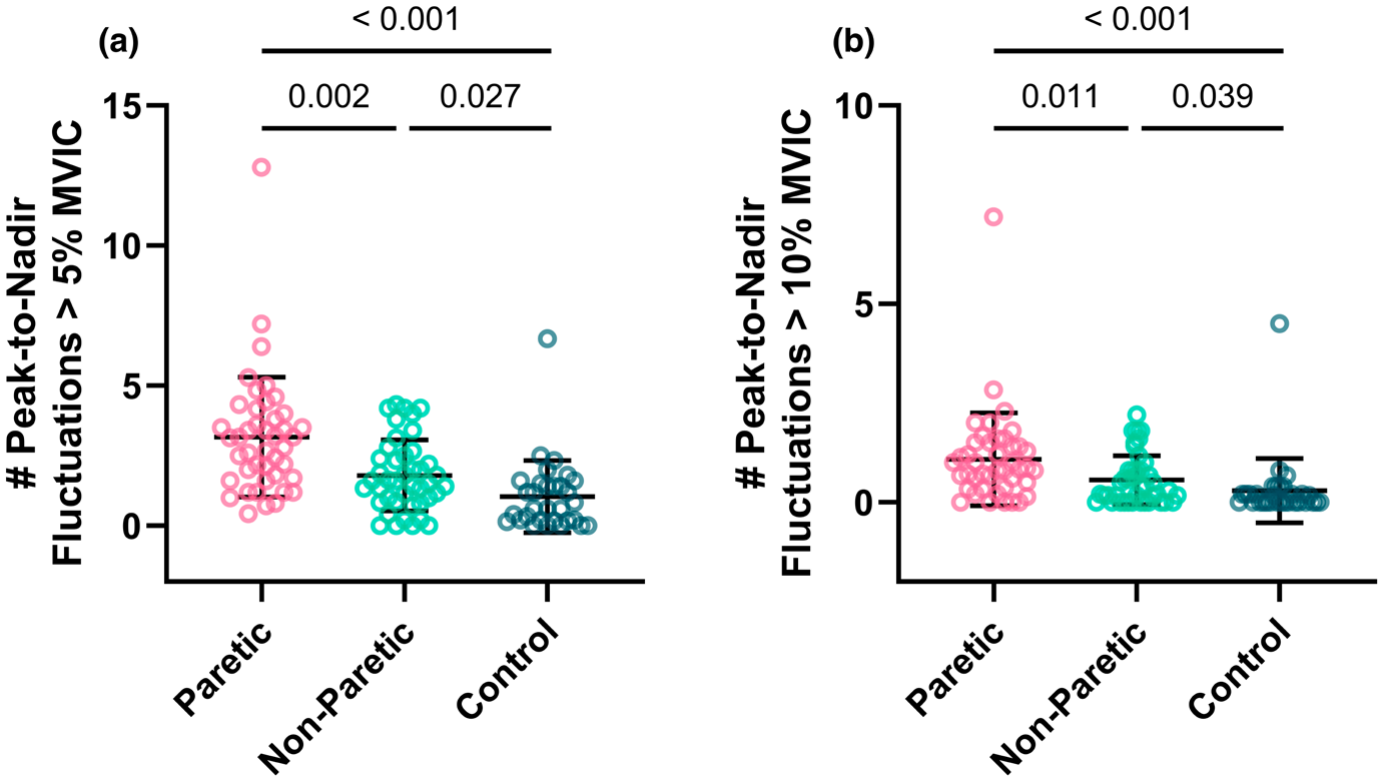
Number of peak‐to‐nadir fluctuations. Both the paretic and non‐paretic legs of stroke survivors had a higher number of peak‐to‐nadir fluctuations that were more than (a) 5% and (b) 10% of the maximal torque value within each maximal voluntary isometric contraction (MVIC) attempt than the dominant leg of neurotypical controls. The paretic leg of the stroke survivors had a higher number of peak‐to‐nadir fluctuations that were more than (a) 5% and (b) 10% of the maximal torque value within each MVIC attempt than their non‐paretic leg.

The number of peak‐to‐nadir fluctuations that were more than 5% and 10% of the maximal torque value within each MVIC attempt are shown in Figure 4. The Kruskal‐Wallis test showed that the differences in the number of peak‐to‐nadir fluctuations that were more than 5% of the maximal torque value within each MVIC attempt among the three testing leg groups were significant (*H* (2) = 33.010, *p* < 0.001). Post hoc pairwise comparisons showed that both the paretic leg (3.17 ± 2.14, *p* < 0.001) and non‐paretic leg (1.79 ± 1.27, *p* = 0.027) of stroke survivors had a higher number of peak‐to‐nadir fluctuations that were more than 5% of the maximal torque value within each MVIC attempt than the dominant leg of neurotypical controls (1.04 ± 1.29). The paretic leg of the stroke survivors had a higher number of peak‐to‐nadir fluctuations that were more than 5% of the maximal torque value within each MVIC attempt than their non‐paretic leg (*p* = 0.002). Similar results held true for the number of peak‐to‐nadir fluctuations that were more than 10% of the maximal torque value within each MVIC attempt (*H* (2) = 26.761, *p* < 0.001). In summary, both the paretic leg and non‐paretic leg of stroke survivors had greater MVIC unsteadiness from the perspective of peak‐to‐nadir fluctuations within each attempt.

### 3.6 *Q*_tw_ inconsistency

**FIGURE 5:**
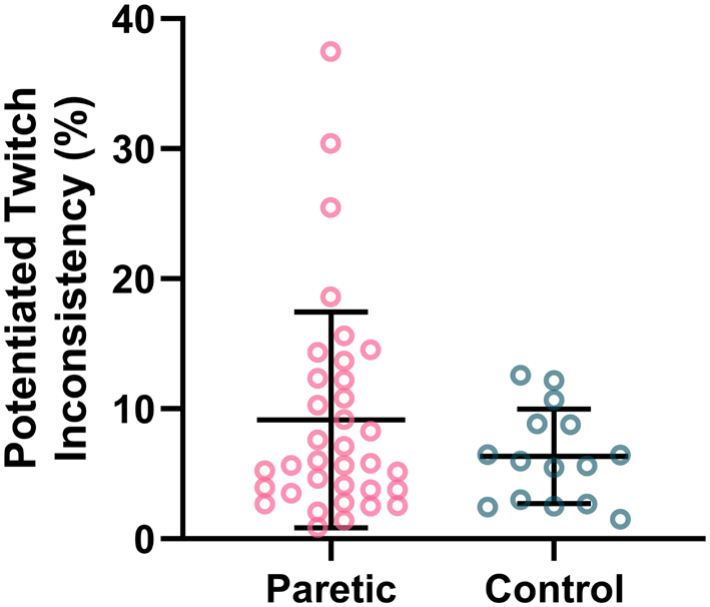
Potentiated twitch (*Q*_tw_) inconsistency (coefficient of variation between the maximal torque values of each *Q*_tw_). There was no significant difference in *Q*_tw_ inconsistency between the paretic leg of stroke survivors and the dominant leg of neurotypical controls.

Results of the *Q*_tw_ inconsistency across discrete attempts are shown in Figure 5. *Q*_tw_ was successfully assessed in a subset of 35 stroke survivors (23 female, paretic leg) and 15 neurotypical controls (9 female, dominant leg). Data were excluded when participants were not able to quickly relax their knee extensor muscles after an MVIC attempt and before the electrical stimulation. There was no significant difference in *Q*_tw_ inconsistency between the paretic leg of stroke survivors and the dominant leg of neurotypical controls (stroke survivors' paretic leg: 9.15 ± 8.31% vs. neurotypical controls' dominant leg: 6.35 ± 3.62%, *U* = 227, *p* = 0.452).

### 3.7 Associations between MVIC variability and motor performance

**FIGURE 6:**
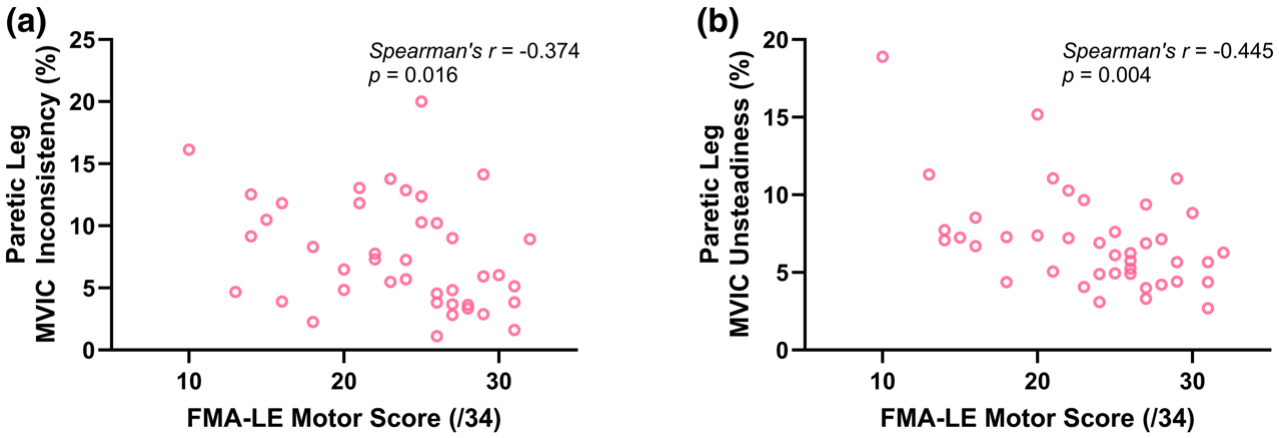
Associations between (a) maximal voluntary isometric contraction (MVIC) inconsistency and (b) MVIC unsteadiness of stroke survivors' paretic leg and their Fugl‐Meyer Assessment‐Lower Extremity (FMA‐LE) motor scores.

In stroke survivors, both MVIC inconsistency (*r*_*s*_ = −0.374, *p* = 0.016, Figure 6a) and MVIC unsteadiness (*r*_*s*_ = −0.445, *p* = 0.004, Figure 6b) of their paretic leg were negatively associated with their FMA‐LE motor scores. However, neither MVIC inconsistency (*r*_*s*_ = −0.121, *p* = 0.439) nor MVIC unsteadiness (*r*_*s*_ = −0.254, *p* = 0.101) was significantly associated with self‐selected walking speed.

### 3.8 MVIC performance over time (first 3 attempts versus last 3 attempts)

**FIGURE 7:**
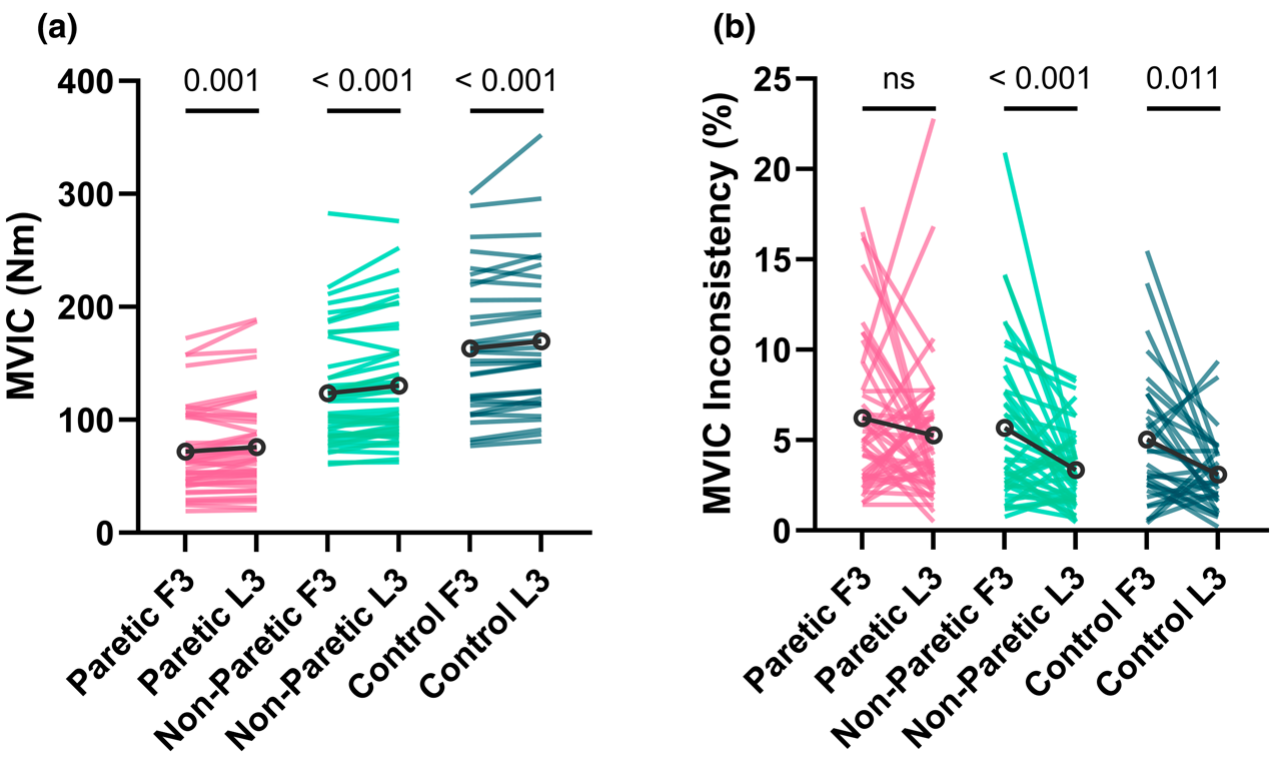
Maximal voluntary isometric contraction (MVIC) performance over time (not followed by electrical stimulation). (a) The paretic and non‐paretic legs of stroke survivors, as well as the dominant leg of neurotypical controls, had higher average maximal torque values during the last 3 (L3) MVIC attempts than the first 3 (F3) MVIC attempts. (b) The non‐paretic leg of stroke survivors and the dominant leg of neurotypical controls had decreased MVIC inconsistency during the L3 MVIC attempts than the F3 MVIC attempts. There was no significant difference in MVIC inconsistency between the F3 and L3 MVIC attempts for the paretic leg of stroke survivors.

As shown in Figure 7a, both the paretic leg (last 3: 75.99 ± 41.42 Nm vs. first 3: 71.74 ± 38.17 Nm, *Z* = 3.272, *p* = 0.001) and the non‐paretic leg (last 3: 130.25 ± 53.45 Nm vs. first 3: 123.48 ± 50.02 Nm, *Z* = 4.166, *p* < 0.001) of stroke survivors had higher average maximal torque values during the last 3 MVIC attempts than the first 3 attempts. Similarly, the dominant leg of neurotypical controls had higher average maximal torque values during the last 3 MVIC attempts than the first 3 attempts (last 3: 169.34 ± 66.24 Nm vs. first 3: 163.29 ± 62.75 Nm, *Z* = 3.488, *p* < 0.001).

As shown in Figure 7b, the non‐paretic leg of stroke survivors (last 3: 3.34 ± 2.14% vs. first 3: 5.68 ± 4.32%, *Z* = −3.429, *p* < 0.001) and the dominant leg of neurotypical controls (last 3: 3.09 ± 2.09% vs. first 3: 5.04 ± 3.76%, *Z* = −2.528, *p* = 0.011) had decreased MVIC inconsistency during the last 3 attempts than the first 3 attempts. There was no significant difference in MVIC inconsistency between the first 3 and last 3 attempts with the paretic leg of stroke survivors (last 3: 5.26 ± 4.10% vs. first 3: 6.22 ± 4.28%, *Z* = −1.107, *p* = 0.268).

For MVIC unsteadiness, none of the three testing leg groups had a significant change from the first 3 to last 3 attempts (stroke paretic leg: *Z* = −1.032, *p* = 0.302; stroke non‐paretic leg: *Z* = −1.854, *p* = 0.064; control dominant leg: *Z* = −1.107, *p* = 0.268).

## 4 DISCUSSION

The primary novel finding of this study was that stroke impacts both dimensions of MOV during knee extension MVICs. Specifically, we found that (1) the paretic leg of stroke survivors had greater inconsistency than the dominant leg of neurotypical controls (Figure 3b) and (2) both the paretic and non‐paretic legs of stroke survivors had greater unsteadiness than the dominant leg of neurotypical controls (Figures 3c and 4). These results support our primary hypothesis that MOV is increased post stroke. In addition, because both neural and muscular mechanisms can contribute to MVIC variability, no significant differences in Q_tw_ inconsistency (muscular mechanisms) between the paretic leg of stroke survivors and the dominant leg of neurotypical controls (Figure 5) suggest potential neural mechanisms for the greater MVIC inconsistency for the paretic leg of stroke survivors. Moreover, we found that both MVIC inconsistency and unsteadiness with stroke survivors' paretic leg were negatively associated with their FMA‐LE motor scores (Figure 6), indicating that stroke survivors with greater stroke‐related motor impairments had greater MOV. Lastly, MVIC inconsistency decreased with practice within the same session for both the non‐paretic leg of stroke survivors and the dominant leg of neurotypical controls but did not for the paretic leg of stroke survivors (Figure 7b).

### 4.1 Decreased MVIC post stroke

Results from the present study show that stroke survivors had a lower knee extension MVIC with their paretic leg versus their non‐paretic leg and the dominant leg of neurotypical controls (Figure 3a). This is consistent with previous findings on various lower extremity muscle groups in stroke survivors (Dorsch et al., 2016; Durand et al., 2015; Fröhlich‐Zwahlen et al., 2014; Hyngstrom et al., 2014; Klein et al., 2010, 2013; Knorr et al., 2011; Murphy et al., 2019; Rybar et al., 2014). This decrease in maximal strength in the paretic limb of stroke survivors is likely due to (1) decreased descending neural drive caused by the damage to the corticospinal tract (Klein et al., 2010; Klein et al., 2013; Knarr et al., 2013) and, to a lesser extent, (2) stroke‐related muscular changes (e.g., atrophy, fiber type switching) (Azzollini et al., 2021; Sions et al., 2012).

### 4.2 Potential mechanisms for increased MOV post stroke

Several factors can contribute to the greater MOV observed in the stroke survivors, especially with their paretic leg. First, it has been shown that stroke‐related damage to the corticospinal tract could result in a decreased ability to individuate joint movement of the arm (McPherson et al., 2018). Relevant to the current study, stroke may also result in greater synergy patterns in the leg and thus greater knee extension MVIC variability. To perform the knee extension MVIC, participants were instructed to work against the flexion synergy pattern as they were trying to contract their knee extensor muscles with both knee and hip joints flexed. It has been shown that it is more challenging for stroke survivors to generate joint torques outside the synergy patterns compared with neurotypical controls (Beer et al., 1999; Dewald & Beer, 2001; Ellis et al., 2007; Sánchez et al., 2018), but improvement could be achieved through neurorehabilitation (Ellis et al., 2009).

Second, inconsistent motor preparation and descending command post stroke could result in differing movement planning and execution for the same task, thus greater inconsistency between attempts. Churchland and colleagues showed that preparatory variability (neural source) contributes to at least 50% of the movement variability during a reaching task in monkeys (Churchland et al., 2006). Similarly, our results suggest that the greater MVIC inconsistency in the paretic leg of stroke survivors was likely attributed to neural versus muscular mechanisms. Because the electrical stimulation to elicit *Q*_tw_ was applied when participants were at rest, the *Q*_tw_ measures primarily provide information distal to the neuromuscular junction (muscular mechanisms) in the absence of volitional effort. As shown in Figure 5, there were no significant differences in *Q*_tw_ inconsistency, thus neural mechanisms may contribute more to the greater MVIC inconsistency in the paretic leg of stroke survivors. Further, although motor preparation differs between goal‐reaching tasks and performing MVICs, preparatory variability may still play a role in the greater MVIC inconsistency in the paretic leg of stroke survivors. Third, stroke survivors often experience sensory deficits on their paretic side, which could interfere with their ability to gauge the magnitude of muscle torque generated and contribute to the greater MVIC variability.

Similar to previous studies (Chow & Stokic, 2011; Hyngstrom et al., 2014; Pellegrino, Coscia, Giannoni, et al., 2021; Pellegrino, Coscia, Pierella, et al., 2021), we observed greater unsteadiness during each MVIC attempt in both the paretic and non‐paretic (to a lesser extent) legs of stroke survivors (Figures 3c and 4). Although we did not test directly, this is likely due to altered motor unit discharge patterns post stroke (Hu et al., 2016; Li et al., 2015; Murphy et al., 2015, 2019). Previous studies have shown that stroke survivors' paretic muscles often had lower motor unit firing rates and higher coefficient variation of motor unit firing rates than neurotypical controls during exercise, which may in part explain the higher MOV that we observed in the current study. In addition, others have shown that stroke modifies the spinal maps and the muscle synergies in both the paretic and non‐paretic limbs but to a different extent (Pellegrino, Coscia, Giannoni, et al., 2021; Pellegrino, Coscia, Pierella, et al., 2021). Spinal maps represent the spatiotemporal organization of electromyography signals at the level of spinal cord, which has been introduced as a possible tool to characterize the output activity of spinal cord to muscles and explore muscle organization. Relevant to the current study, Pellergrino et al. and colleagues investigated spinal maps and muscle synergies during an isometric task, and they demonstrated that stroke affected the ability of the non‐paretic arm, though to a minor extent compared to the paretic arm, to produce isometric force (Pellegrino, Coscia, Giannoni, et al., 2021). Although we focused on knee extensor muscles in the current study, spinal maps and muscle synergies are likely to be altered by stroke as well for both paretic and non‐paretic legs, which may also in part explain why we observed greater MVIC unsteadiness on both legs of stroke survivors.

### 4.3 Increased MOV post stroke and functional performance

This study showed that stroke survivors had greater inconsistency and unsteadiness during knee extension MVIC with their paretic leg and these greater MOV measurements were moderately associated with greater motor impairments assessed using the FMA‐LE (Figure 6). This is consistent with previous literature that greater unsteadiness during submaximal isometric contractions is associated with poorer performance in functional tests (e.g., walking, balance, and dexterity) in older neurotypical adults and people with neurological conditions (Almuklass et al., 2018; Chow & Stokic, 2011; Christou et al., 2003; Christou & Enoka, 2011; Davis et al., 2020; Hyngstrom et al., 2014; Lodha et al., 2016).

### 4.4 Within‐session performance across repeated attempts

As shown in Figure 7a, stroke survivors (both paretic and non‐paretic legs) and neurotypical controls (dominant leg) were able to produce a greater average MVIC torque during the last 3 attempts compared with the first 3 attempts; however, we only observed decreased inconsistency, from the first 3 to last 3 attempts, for the dominant leg of neurotypical controls and the non‐paretic leg, but not the paretic leg, of stroke survivors (Figure 7b). Over time with practice and repeated attempts within the same session, stroke survivors were able to generate more torque with their paretic knee extensor muscles, but there was no detectable improvement in MVIC inconsistency or unsteadiness. These results suggest that the mechanisms for potential improvement in MVIC variability and those driving the increase in MVIC torque (e.g., rate coding and recruitment of motor units) are likely not the same and need to be studied in the future.

### 4.5 Implications for stroke assessment and rehabilitation

The present study identified the impairments in MOV among stroke survivors, particularly with their paretic leg, during knee extension MVIC measurements that might not improve with practice within the same session. The accuracy of maximal muscle strength measurements is important because maximal muscle strength informs critical information when assessing function post stroke and evaluating rehabilitation progress. Although manual muscle testing (MMT) has been widely used in clinical settings, the ordinal nature of the MMT's grade may make it difficult to quantify rehabilitation progress. Thus, coupling MMT and dynamometer assessments should be considered clinically. Our results support the use of multiple attempts when measuring maximal muscle strength through MVIC and may pave the way for longitudinal studies to compare MOV between testing sessions during rehabilitation programs and evaluate the effectiveness of the intervention for stroke survivors.

### 4.6 Experimental considerations and future directions

First, our small sample size within a specific population of stroke survivors limited our ability to generalize these findings to all stroke survivors. Future longitudinal studies will include a larger sample size and assess stroke survivors throughout the acute, subacute, and chronic stages of stroke recovery. Second, we focused on the *Q*_tw_ measurements to understand muscular mechanisms for the increased MOV. Future studies will investigate neural mechanisms for increased MOV in stroke survivors. Third, the testing order of the legs was not randomized in stroke survivors, and this could be a limitation because of the within‐leg learning effect as we observed in the current study. This was because we wanted the participants to give their best performance with their paretic leg, and testing the non‐paretic leg first may result in potential neural fatigue, which might limit the performance of their paretic leg. Also, given that we still showed greater MVIC inconsistency for the non‐paretic leg of stroke survivors than the dominant leg of neurotypical controls, the between‐leg learning effect may not be as apparent. Additionally, while we recognize that MOV is complex and often involves multiple joints, the MVIC task in the current study was a single‐joint exercise. Future studies will investigate MOV during more complex tasks that involve multiple joints and/or multiple muscle groups with surface electromyography assessment to explore the potential effects of co‐contraction between the agonist and antagonist muscles on MOV.

### 4.7 Conclusions

This study showed that, compared with neurotypical controls, stroke survivors had greater MOV (inconsistency and unsteadiness) during a single session of MVIC measurements with multiple attempts, and the greater MVIC MOV was moderately associated with poorer clinical measures of motor performance. We observed no differences in *Q*_tw_ inconsistency between stroke survivors and neurotypical controls, indicating that the greater MVIC inconsistency is likely driven by neural mechanisms. These results highlight the importance of using multiple attempts when assessing maximal muscle strength in stroke survivors.

## AUTHOR CONTRIBUTIONS

**Zhilun Zhou:** Conceptualization; data curation; formal analysis; methodology; validation; visualization. **Stephanie C. Wolfe:** Conceptualization; data curation. **Brian D. Schmit:** Methodology. **Matthew J. Durand:** Funding acquisition. **Sandra K. Hunter:** Conceptualization. **Allison S. Hyngstrom:** Conceptualization; funding acquisition; project administration; supervision.

## FUNDING INFORMATION

This work was supported by a *Eunice Kennedy Shriver* National Institute of Child Health and Human Development Grant (R01HD099340, to Allison S. Hyngstrom and Matthew J. Durand), a National Institute on Aging Predoctoral to Postdoctoral Fellow Transition Award (F99AG083283 & K00AG083283, to Zhilun Zhou), and an American Heart Association Predoctoral Fellowship (903373, to Zhilun Zhou).

## CONFLICT OF INTEREST STATEMENT

The authors declare no conflicts of interest.

## ETHICS STATEMENT

All procedures in the current study were approved by Marquette University Institutional Review Board (HR‐1812027206). A written informed consent was obtained from each participant before any study activities.

## Data Availability

Data will be made available upon reasonable request to the corresponding author.
